# A culturally adapted manual-assisted problem-solving intervention (CMAP) for adults with a history of self-harm: a multi-centre randomised controlled trial

**DOI:** 10.1186/s12916-023-02983-8

**Published:** 2023-07-31

**Authors:** Nusrat Husain, Tayyeba Kiran, Imran Bashir Chaudhry, Christopher Williams, Richard Emsley, Usman Arshad, Moin Ahmed Ansari, Paul Bassett, Penny Bee, Moti Ram Bhatia, Carolyn Chew-Graham, Muhammad Omair Husain, Muhammad Irfan, Ayesha Khaliq, Fareed A. Minhas, Farooq Naeem, Haider Naqvi, Asad Tamizuddin Nizami, Amna Noureen, Maria Panagioti, Ghulam Rasool, Sofiya Saeed, Sumira Qambar Bukhari, Sehrish Tofique, Zainab F. Zadeh, Shehla Naeem Zafar, Nasim Chaudhry

**Affiliations:** 1 Division of Psychology and Mental Health, University of Manchester, Oxford Rd, Manchester, M13 9PL England UK; 2 Mersey Care NHS Foundation Trust, Kings Business Park, Trust Offices/V7 Buildings, Prescot, L34 1PJ England UK; 3 Pakistan Institute of Living and Learning, Suite No. 201, 2nd Floor, The Plaza, Do-Talwar, Khayaban-E-Iqbal, Clifton, Karachi, Pakistan; 4 Department of Psychiatry, Ziauddin University and Hospital, 4/B Shahrah-E-Ghalib Rd, Block 6 Clifton, Karachi, Pakistan; 5 Institute of Health and Well Being, University of Glasgow, Glasgow, G12 8QQ Scotland UK; 6 Medical Statistics & Trials Methodology, Institute of Psychiatry, Kings College London, Strand, London, WC2R 2LS England UK; 7 Liaquat University of Medical and Health Sciences, C7PC+337, Hyderabad, Jamshoro Pakistan; 8 Statistical Consultancy, Hemel Hempstead, England UK; 9 Division of Nursing, Midwifery and Social Work, University of Manchester, Oxford Rd, Manchester, M13 9PL England UK; 10 Peoples University of Medical & Health Science for Women Nawabshah, 6CV3+7HW, Hospital Road, Shaheed Benazirabad, Nawabshah, Pakistan; 11 School of Medicine, Keele University, Newcastle, ST5 5BG Keele UK; 12 Department of Psychiatry, University of Toronto, 399 Bathurst St, Toronto, ON M5T 2S8 Canada; 13 Department of Mental Health, Psychiatry and Behavioural Sciences, Peshawar Medical College, Riphah International University, Islamabad, Pakistan; 14 Rawalpindi Medical University, Rawalpindi, Pakistan; 15 CAMH, University of Toronto, 399 Bathurst St, Toronto, ON M5T 2S8 Canada; 16 Department of Psychiatry, Dow University of Health Sciences, Mission Rd, New Labour Colony Nanakwara, Karachi, Pakistan; 17 Institute of Psychiatry, Benazir Bhutto Hospital, Near Chandni Chowk, Murree Rd, Chah Sultan, Rawalpindi, Pakistan; 18 Division of Population Health, Health Services Research & Primary Care, Institute for Health Policy and Organisation/Alliance Manchester Business School, University of Manchester, Oxford Rd, Manchester, M13 9PL UK; 19 Balochistan Institute of Psychiatry & Behavioural Sciences, Bolan Medical College, 5XRG+VGC, Brewery Rd, Quetta, Pakistan; 20 Department of Psychiatry, Services Institute of Medical Sciences, G8QM+JWR, Jail Rd, Shadman 1 Shadman, Lahore, Pakistan; 21 Institute of Nursing, Iqra University, G-16/1 Allama Rasheed Turabi Rd, Block-B Block B, North Nazimabad Town, Karachi, Pakistan

**Keywords:** Suicide prevention, Self-harm, CMAP, Cognitive behaviour therapy, Problem-solving; Low-income setting, RCT

## Abstract

**Background:**

Self-harm is an important predictor of a suicide death. Culturally appropriate strategies for the prevention of self-harm and suicide are needed but the evidence is very limited from low- and middle-income countries (LMICs). This study aims to investigate the effectiveness of a culturally adapted manual-assisted problem-solving intervention (CMAP) for patients presenting after self-harm.

**Methods:**

This was a rater-blind, multicenter randomised controlled trial. The study sites were all participating emergency departments, medical wards of general hospitals and primary care centres in Karachi, Lahore, Rawalpindi, Peshawar, and Quetta, Pakistan. Patients presenting after a self-harm episode (*n* = 901) to participating recruitment sites were assessed and randomised (1:1) to one of the two arms; CMAP with enhanced treatment as usual (E-TAU) or E-TAU. The intervention (CMAP) is a manual-assisted, cognitive behaviour therapy (CBT)-informed problem-focused therapy, comprising six one-to-one sessions delivered over three months. Repetition of self-harm at 12-month post-randomisation was the primary outcome and secondary outcomes included suicidal ideation, hopelessness, depression, health-related quality of life (QoL), coping resources, and level of satisfaction with service received, assessed at baseline, 3-, 6-, 9-, and 12-month post-randomisation. The trial is registered on ClinicalTrials.gov. NCT02742922 (April 2016).

**Results:**

We screened 3786 patients for eligibility and 901 eligible, consented patients were randomly assigned to the CMAP plus E-TAU arm (*n* = 440) and E-TAU arm (*N* = 461). The number of self-harm repetitions for CMAP plus E-TAU was lower (*n* = 17) compared to the E-TAU arm (*n* = 23) at 12-month post-randomisation, but the difference was not statistically significant (*p* = 0.407). There was a statistically and clinically significant reduction in other outcomes including suicidal ideation (− 3.6 (− 4.9, − 2.4)), depression (− 7.1 (− 8.7, − 5.4)), hopelessness (− 2.6 (− 3.4, − 1.8), and improvement in health-related QoL and coping resources after completion of the intervention in the CMAP plus E-TAU arm compared to the E-TAU arm. The effect was sustained at 12-month follow-up for all the outcomes except for suicidal ideation and hopelessness. On suicidal ideation and hopelessness, participants in the intervention arm scored lower compared to the E-TAU arm but the difference was not statistically significant, though the participants in both arms were in low-risk category at 12-month follow-up. The improvement in both arms is explained by the established role of enhanced care in suicide prevention.

**Conclusions:**

Suicidal ideation is considered an important target for the prevention of suicide, therefore, CMAP intervention should be considered for inclusion in the self-harm and suicide prevention guidelines. Given the improvement in the E-TAU arm, the potential use of brief interventions such as regular contact requires further exploration.

## Background

The World Health Organisation (WHO) reported that there are more than 700,000 suicide deaths worldwide in 2019 [1]. More than 77% of suicide deaths are in low and middle-income countries (LMICs) [1]. Self-harm is an important predictor of suicide death, typically with more than 20 attempts prior to suicide [2]. More than 39% of all suicides globally occur in South Asia [3]. However, these rates are underreported evidenced by a verbal autopsy study from a South Asian setting (India) where suicide rates were underestimated by 25% for men and 36% for women compared to the official data [4].

There are no official suicide data from Pakistan [5]. Both self-harm and suicide were considered illegal acts until recently (December 2022) when a bill was passed by the Senate abolishing the provision of punishment for those who attempt suicide, an important step towards preventing suicide [6]. Self-harm is socially and religiously condemned in Pakistan [7]. A family’s fear of the community grapevine and the perceived negative impact of self-harm and suicide on the family’s honour (izzat) has been reported by clinicians as a major barrier to help-seeking in Pakistan [7]. The problems are further exacerbated by a lack of awareness about the role of psychological services and social stigma [7]. Self-harm is reported as a consequence of a complex interplay of multiple factors including severe mental health problems, financial difficulties, interpersonal conflicts with family, and poor problem-solving abilities [7]. Service level challenges have also been reported including limited access to psychological services and a lack of training arrangements for health professionals such as general practitioners and emergency care staff [7, 8].

The WHO (2021) has recommended a public health approach to identify and provide treatment to high-risk individuals, particularly those with a history of self-harm. There is established evidence on the management of self-harm in high-income countries [9], but there are no national recommendations for the prevention and treatment of self-harm in Pakistan. Psychosocial interventions help people at risk of suicide by addressing the underlying psychological risk factors associated with self-harm, for example by helping people improve their coping skills and solve specific problems more effectively, manage psychiatric disorders such as depression, improve self-esteem, increase a sense of social connectedness, and reduce impulsivity and harmful reactions to distressing situations [10]. Cognitive behaviour therapy (CBT) based psychological interventions help people evaluate ways in which they interpret a stressful situation and offer them support in changing how they deal with problems [5, 10, 11]. Problem-solving therapy is an integral part of CBT, that can be delivered as a therapy in itself [10]. A recent Cochrane review of randomised controlled trials (RCTs) on psychosocial interventions for the prevention of self-harm in the adult population has highlighted that most of the trials (*n* = 20) investigated the role of individually delivered CBT-based psychotherapy compared to limited trials on Dialectical Behaviour Therapy (DBT) (*n* = 6), Mentalisation-Based Therapy (MBT) (*n* = 1) and Emotion Regulation Psychotherapy (*n* = 2) [10]. This review reports beneficial effects for CBT-based psychological approaches at longer follow-up time points, and beneficial effects for MBT, and emotion-regulation psychotherapy at the post-intervention assessment, though these results warrant further investigation because of low to moderate level of certainty of evidence. The National Institute for Health and Care Excellence (NICE) guidelines have identified the potential role of CBT-based psychotherapy that is specifically tailored for adults who self-harm in prevention of self-harm repetition [12].

*C*ulturally adapted manual-*a*ssisted *p*roblem-solving intervention (CMAP) is a CBT-based intervention that has been evaluated in Pakistan in a randomised controlled trial (RCT) with adult self-harm survivors (*n* = 221) recruited from medical units in Karachi (the most populous city in Pakistan) [5]. The intervention (CMAP) was adapted (for the cultural adaptation process please see method section) from a CBT-based self-help guide called “Life after self-harm” [13]. The CMAP intervention utilises problem-solving components within a brief CBT intervention that can be widely utilised in clinical practice and also includes other components such as a session on harm minimisation by developing a crisis plan and involving family members and carers advised by NICE guidelines [12]. Since most episodes of self-harm in Pakistan are precipitated by interpersonal problems with family members, there is a strong rationale for investigating the effectiveness of an intervention which addresses such issues. In addition, CMAP is a structured intervention that is briefer than many existing CBT programmes for self-harm, facilitating its implementation within low-income countries by minimising demands on staff and services, and brief interventions in low- and middle-income countries (LMICs) have been found to be effective in reducing the number of suicide deaths [14]. The main outcome measures in this exploratory study were suicidal ideation, the severity of depression and hopelessness assessed at baseline, 3 and 6 months. There was a significant reduction from baseline in suicidal ideation, the severity of depression and hopelessness in the CMAP arm compared to the treatment-as-usual (TAU) arm at each follow-up assessment. Though the results were encouraging, the sample size was small to provide a definitive answer. Furthermore, this exploratory RCT addressed short-term outcomes to 6 months only, with participants recruited from 3 general hospitals in one city. Patients who were not admitted to the medical wards were excluded as the research team did not have the resources to include this group. All of these limitations were addressed in this current, large-scale definitive RCT of the same intervention (CMAP) added to the enhanced treatment as usual (E-TAU) compared to the E-TAU alone, for reducing repeat self-harm episodes, and several other clinical and health outcomes 12-month post-randomisation among adults presenting after episode of self-harm in five large cities across Pakistan.

## Methods

### Study design

The study was a multicenter, randomised controlled trial with randomisation of individual patients into either of two arms: (1) CMAP plus E-TAU and (2) E-TAU alone. The trial is reported in accordance with the guidance of the Consolidated Standards of Reporting Trials (CONSORT).

### Study setting

Study sites were all participating emergency departments, medical wards of general hospitals and primary care centres in Karachi (population 21 million), Lahore (12 million), Rawalpindi (4.7 million), Peshawar (1.9 million), and Quetta (1 million), Pakistan.

### Participants

The target population was all adults presenting to recruitment sites after self-harm episodes.

#### Inclusion criteria

In this trial’s context, self-harm was defined as:

“an act with non-fatal outcome, in which an individual deliberately initiates a non-habitual behaviour that, without interventions from others, will cause self-harm, or deliberately ingests a substance in excess of the prescribed or generally recognised therapeutic dosage, and which is aimed at realising changes which the subject desired via the actual or expected physical consequences [5, 15]”.

1. Individuals aged 18 years and above.
2. Residents of the catchment area of participating recruitment centres.
3. Individuals not requiring inpatient psychiatric treatment, as determined by clinical teams.

#### Exclusion criteria

1. Temporary resident with less likelihood of availability for follow-up.
2. Participants with serious general medical conditions, substance misuse, delirium, dementia, alcohol or drug dependence, bipolar disorder, schizophrenia, and learning disabilities, as determined by clinical teams.
3. Not able to engage, participate and/or respond to the trial questionnaires due to a medical or psychiatric condition, or due to living outside of the study catchment area.

### Randomisation and masking

The completed baseline assessments were sent by the trained researchers to the Trial Manager who then contacted the off-site randomisation centre, where eligibility was re-checked, baseline measures recorded and participant trial numbers assigned. Treatment assignment was determined using block randomisation controlling for gender, age, and type of self-harm behaviour. For the block randomisation, study site, age group (> 30 or <  = 30), gender and type of self-harm were included as strata. However, self-harm was only included as any chemical (including bleach and pesticides) or other. A randomisation list was generated using online resource-sealed envelopes. The online resource was used with block sizes 2, 4, 6 and 8.

The off-site statistician and research team carrying out follow-up assessments were blinded to treatment allocation. Trial participants and therapists were not blinded to treatment allocation as evidence suggests that blinding of participants and therapists may compromise the effects of the active ingredients of the psychological intervention. Effective delivery of a particular psychological intervention requires extensive training, which would be difficult to implement with the blinding intact [16].

### Study procedure

All procedures contributing to this work comply with the Helsinki Declaration of 1975, as revised in 2008. The study was approved by the Research Ethics Committee of the Karachi Medical and Dental College (027/15) and the University of Manchester (2019–2610-10693). The study's clinical trial registration number is NCT02742922 — registered with ClinicalTrials.gov.

All patients presenting to the participating sites following an episode of self-harm were approached for recruitment. Detailed information about the research along with a Participant Information Leaflet was given to the potential participants. Potential participants were assessed by trained researchers against study eligibility criteria. Handwritten signatures (or thumbprints) were used to obtain informed consent from eligible participants. A trained researcher scheduled time with consented participants and baseline assessments were completed face to face either at a research office or the participant’s home. Following baseline assessments, a unique identification number (ID) was assigned to each participant and a list of IDs with details on age, gender, method of self-harm and study site was prepared by the trial manager and sent for randomisation. All the participants were made aware of their respective treatment arm within 1 week of randomisation. Participants in the intervention arm were contacted by the therapist to arrange the first session. All intervention sessions were delivered face to face either at a research office or the participant’s home, at a time convenient for both the therapist and participant. Follow-up assessments with participants from both study arms were carried out at 3-, 6-, 9-, and 12-month post-randomisation. All follow-up assessments were carried out face to face either at a research office or the participant’s home.

### Intervention

CMAP is a manual-assisted, CBT-informed problem-focused therapy, comprising six one-to-one sessions delivered over 3 months. This has been culturally adapted and refined with permission from a self-help guide “Life after self-harm” [13]. Intervention includes an in-depth understanding of the self-harm episodes such as discussion on triggers of self-harm episodes, the reaction of family members, crisis management for risk minimisation, problem-solving skills, CBT techniques to manage negative thinking and emotions, and strategies for relapse prevention. The last session was with the family to discuss their emotions related to the self-harm episodes, encouraging them to seek professional help if they observe any further risk of self-harm episodes. The intervention was delivered at a place of the participant’s choice (the participant’s home or an outpatient clinic/research office). The first 2 sessions were delivered weekly, and then fortnightly. Each session lasted for about 50 min.

### Cultural adaptation

Before the exploratory study, a group of mental health professionals translated the content of the manual into Urdu (Pakistan’s national language). A focus group with multidisciplinary health professionals (mental health professionals, general physicians, nurses) was conducted to discuss cultural adaptations, and special consideration was given to phrases and concepts to reflect Pakistani culture. Additionally, culturally appropriate case scenarios were incorporated and a consensual view to addressing cultural factors such as gender role, family conflicts and financial difficulties was taken. Issues related to substance misuse were replaced with more emphasis on family conflicts (culturally sensitive training in assertiveness and conflict management) as these conflicts usually lead to a self-harm episode in Pakistan.

### Enhanced treatment as usual (E-TAU)

Local primary care, psychiatric and medical services offer standard care according to available resources. People who self-harm would not be routinely referred to psychiatric facilities. Along with TAU, participants in the E-TAU arm received full assessments at baseline, 3, 6, 9, and 12 months, in addition to a monthly call from a designated researcher to ensure their ongoing engagement with the project.

### Assessments

#### Demographic questionnaire

This was a structured form specifically prepared for the study to collect demographic information (age, sex, education, etc.).

### Primary outcome measure

#### Suicide Attempt Self-Injury Interview (SASII) [17]

Repetition of self-harm episodes at 12-month post-randomisation were recorded using the semi-structured questionnaire SASII. Information was collected about the method, time, antecedents, functions and circumstances leading to self-harm. SASII has good validity and inter-rater reliability (ICC = 0·96) [17].

### Secondary outcome measures

#### Beck Scale for Suicide Ideation (BSI) [18]

This is a self-report questionnaire (19 items) to assess the severity of suicidal ideation in the previous week. Scores range from 0 to 38 and higher scores on the questionnaire (≥ 6) suggest a greater risk of suicide [19]. No specific cut-off scores exist to classify severity; however, higher scores reflect greater suicide risk. The Urdu-translated version has a Cronbach’s alpha of 0.89 [20].

#### Beck Hopelessness Scale (BHS) [21]

This is a 20-item self-report assessment of hopelessness, feelings about the future and loss of motivation. Scores range between 0 and 20. Higher scores indicate increasing severity of hopelessness: 0–3 minimal, 4–8 mild, 9–14 moderate, and 15–20 severe. The reliability coefficient of the Urdu version is 0.93 [20].

#### Beck Depression Inventory (BDI) [22]

This is a 21-item instrument to assess depressive symptoms. A higher score indicates greater severity of depression. A score between 1 and 10 indicates that the ups and downs are considered normal, 11 and 16 mild mood disturbance, 17 and 20 borderline clinical depression, 21 and 30 moderate depression, 31 and 40 severe depression and a score above 40 indicate extreme depression. The Cronbach’s alpha of the Urdu-translated version was 0.97 [20].

#### Coping Resource Inventory (CRI) [23]

The CRI is a structured instrument to measure the coping resources available to an individual to deal with stress. The CRI has five domains;

The cognitive domain assesses the extent to which individuals maintain a positive sense of self-worth, a positive outlook towards others, and optimism about life in general. Examples of questions include: “I see myself as lovable”.

The social domain assesses the degree to which individuals are connected to social networks that provide support in stressful times. An example question is: “I am part of a group, other than my family that cares about me”.

The emotional domain assesses the degree to which individuals are able to express a range of emotions. An example question is: “I express my feelings clearly and directly”.

The spiritual/philosophical domain assesses the degree to which actions of an individual are guided by a stable set of values derived from personal philosophy or from familial, religious, or cultural tradition. An example question is: “My values and beliefs help me meet daily challenges”.

The physical domain assesses the degree to which an individual is able to perform health-promoting behaviours that can contribute to increased physical wellbeing. An example question from this domain is: “I exercise vigorously 3–4 times a week”.

A four-point rating scale is used to indicate how often an individual has engaged in the item over the past 6 months. The sums of the item responses for each scale constitute the scale scores. The total resource score is computed by adding the five individual scale scores. The higher the scores, higher is the coping resources of that individual [23].

Psychometric properties of CRI are well-established [23]. Test–retest correlation coefficients ranged from 0.60 to 0.73 and Cronbach’s alpha from 0.77 to 0.91 for the six domains. The predictive, concurrent, and discriminant validity for the scale has been established.

#### EuroQol – 5 Dimensions (EQ-5D) [24]

This is a standardised, self-report questionnaire covering five dimensions of health (mobility, self-care, usual activities, pain/discomfort, and anxiety/depression). Each dimension has responses in 3 levels of intensity: (level 1) no problems, (level 2) some problems, and (level 3) extreme problems. Participants are also asked to provide a self-rating on a Visual Analogue Scale (VAS), ranging from 0 (worst imaginable health state) to 100 (best imaginable health state). The EQ-5D total score is converted into an EQ-5D index score using already established valuation sets. Pakistan does not have a preference-based value set for the EQ-5D-3L instrument; therefore, the Thailand time trade-off tariff was applied. Values, 1–3, were assigned to each level of EQ-5D-3L. Value 1 indicates perfect health, and 3 is the worst in each dimension. In the first step, all the responses of level were mentioned together for each participant such as from (1 1 1 1 1) to (3 3 3 3 3) and in the second step a crosswalk table was used to compute the index score (using Thailand tariff). Test–retest reliability assessments in the general population reported moderately weighted kappa (*k*) (*k*: 0.42–0.63) and high intra-class correlation coefficients (ICCs: 0.78) [25]. Studies with individuals experiencing mental health difficulties reported ICC = 0.83 for common mental disorders such as depression and ICC = 052 for severe mental illnesses such as schizophrenia [26].

#### Client Satisfaction Questionnaire (CSQ) [27]

The CSQ-8 is an unidimensional measure of an individual’s satisfaction with services, assessed at 3 (end of treatment) and 12-month post-randomisation. The CSQ-8 has eight questions: quality of service, kind of service, meet needs, recommend to a friend, amount of help, deal with problems, overall satisfaction, and come back. The individual responds to these questions using a 4-point Likert scale. Their responses are scored from 1 to 4, and the total scores range from 8 to 32. Higher scores indicate greater satisfaction. Reliability testing CSQ-8 reported a high internal consistency score ranging between 0.83 and 0.93 [28].

#### Cognitive Therapy Rating Scale (CTRS) [29]

The CTRs is an observer-rated evaluation of a therapist’s competence in cognitive therapy skills. The questionnaire includes 12 items, scored on a 7-point Likert-type scale ranging from 0 (*poor*) to 6 (*excellent*). Items are designed to assess therapeutic relationship skills (e.g. interpersonal effectiveness), CBT-specific skills (e.g. focusing on key cognitions and behaviours), and structure (e.g. agenda setting). Internal consistency across all items is high (*α* = 0.94) [30].

#### Client Services Receipt Inventory [31]

Information on participants’ use of both formal and informal (such as Imams/faith healers) health services was collected at baseline and follow-up assessments using a structured form. We will submit the economic evaluation as a separate publication.

*Translation:* All Urdu version questionnaires mentioned above have been used before in the exploratory trial [5].

*Adverse events monitoring:* Adverse events were recorded on the adverse event form that was developed for the trial.

### Training and supervision

Researchers were trained by senior mental health professionals in recruiting vulnerable populations (including those with severe mental illnesses), administering both qualitative and quantitative assessments, managing distressed participants, and retaining difficult-to-engage populations. The research team was trained in Good Clinical Practice (GCP), data protection and management, research and information governance. Monthly training refreshers were conducted to ensure the accuracy and concordance of ratings. These trainings involved both live role play and videotaped sessions of mock interviews. All trial therapists also received regular ongoing training. These training sessions included presentations on CMAP, role-play, discussion on how to introduce and get homework assignments completed, and feedback on role-play. All trial therapists also received regular ongoing training and supervision by national CBT therapists (ZZ, SS) as well as international experts (CW, FN). Digitally recorded sessions with the participants were rated by the CBT supervisor (ZZ) using the Cognitive Therapy Rating scale (CTRs) [29].

### Sample size

Based on previous analysis of therapist-delivered intervention trials, we believe that the intra-cluster correlation coefficient (ICC) for therapists [32] is likely to have a value between 0.01 and 0.05 for this type of outcome measure. This trial used repetition of self-harm as its primary outcome measure because it is a strong risk factor of a suicide death. The expected event rates of 27.7% and 16.1% come from the study by Brown et al. [33]. Brown reported a significant difference in the rate of repetition of self-harm over an 18-month period, 24.1% in the cognitive therapy group and 41.6% in usual care. We estimated from this that the event rate would be 27.7% (two-thirds of 41.6%) in 12 months in the usual care arm and 16.1% (two-thirds of 24.1%) in the CMAP arm. Under these assumptions, a sample of 624 randomised patients was required to have 80% power to detect this difference assuming a 5% significance level. However, the funding panel advised consulting an independent statistician (KG) to increase the power to 90% thus increasing the sample size from 624 to 850. We randomised a total of 901 participants as consent was already obtained from participants across different sites.

### Statistical analysis

Statistical analysis was based on intention-to-treat subject to the availability of data. The statistical analysis of the primary outcome measure, repetition of the self-harm episode, was performed using a logistic random effects model, with the therapist included as a random effect. The E-TAU group did not receive trial intervention, and thus for the purposes of the model, each E-TAU participant was considered to be in their own cluster. Also included in the model were adjustments for age, gender, type of self-harm and level of depression at baseline (see Table 2 for sub-groups).

Continuous secondary outcome measures were analysed using a linear mixed model, with a single model fitted with data across all time-points. Both therapist and the patient were included as random effects. Covariates included were for the primary outcome, plus the baseline values of the outcome.

Secondary outcomes measured on an ordinal scale (individual CSQ-8 items) were analysed using an ordinal logistic regression random effects model, with the therapist as the random effect (Table 5). Covariates in the model were as for the primary outcome.

## Results

**Fig. 1:**
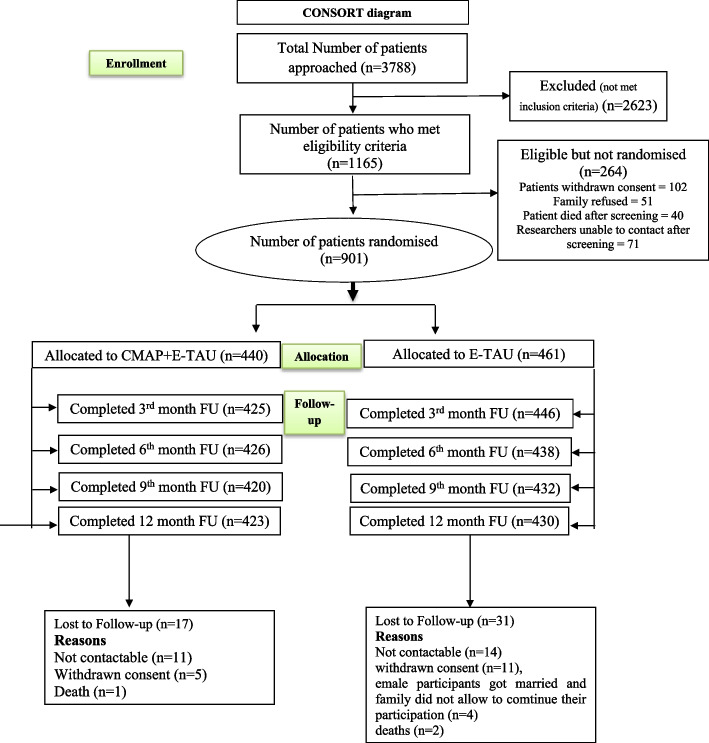
CONSORT diagram

A total of 3788 patients completed initial screening against eligibility criteria and 1165 met trial inclusion criteria. A total of 901 patients were randomised either into the intervention arm (*n* = 440) or the E-TAU arm (*n* = 461). The first participant was randomised on 27th April 2016 (as per the details mentioned on ClinicalTrials.gov) and the last participant on 20th May 2018. Follow-up assessments started in August 2016 and completed in July 2019. A total of 423 (96%) in the intervention arm and 430 (93%) in the E-TAU arm completed 12-month follow-up assessments (please see Fig. 1 CONSORT diagram).

Out of 901 participants, 544 (60.4%) were women, and the mean age of participants was 26.5 years (SD = 7.97); 523 (58%) were married; 474 (52.6%) were from a nuclear family; 464 (51.5%) belonged to ultra-lower income group (earning 147 US dollars/month) and 202 (22.9%) were from the lower middle-income group (160 US dollars and above/month); 284 (31.5%) had received up to 10 years of schooling; and 539 (59.8%) were employed (Table 1). Overall, 457 (50.7%) participants reported they were in debt, 566 (62.8%) participants reported that they had difficulty meeting day-to-day expenses in the last month, and 346 (38.4%) reported they had gone to sleep hungry due to financial difficulties at some point during the past month (Table 1).

**Table 1:** Sociodemographic characteristics of participants by treatment group

	**E-TAU (*N* = 461)**	**CMAP plus E-TAU (*N* = 440)**	**Total (*N* = 901)**
**Gender**			
Male	186 (40.3)	171 (38.9)	357 (39.6)
Female	275 (59.7)	269 (61.1)	544 (60.4)
**Marital status**			
Single	161 (34.9)	144 (32.7)	305 (33.9)
Married	257 (55.7)	266 (60.5)	523 (58.0)
Separated/divorce/widow	43 (9.3)	30 (6.8)	73 (8.1)
**Family status**			
Joint	212 (46.0)	215 (48.9)	427 (47.4)
Nuclear	249 (54.0)	225 (51.1)	474 (52.6)
**Status of home**			
Own	308 (66.8)	300 (68.2)	608 (67.5)
Rent	153 (33.2)	140 (31.8)	293 (32.5)
**Socio economic status**			
Ultra lower income group (monthly earning up to PKR 15,000)	231 (50.1)	233 (53.0)	464 (51.5)
Lower income group (monthly earning between PKR 15,001 to 32,000)	122 (26.5)	109 (24.8)	231 (25.6)
Lower middle-income group (monthly earning PKR 32,001 and above)	108 (23.4)	98 (22.3)	206 (22.9)
**Education**			
No formal education	114 (24.7)	110 (25.0)	224 (24.9)
Up to primary (1–5)	129 (28.0)	117 (26.6)	246 (27.3)
Up to secondary (6–10)	144 (31.2)	140 (31.8)	284 (31.5)
Up to 12 years (11–12)	70 (15.2)	68 (15.5)	138 (15.3)
Above 12 years	4 (0.9)	5 (1.1)	9 (1.0)
**Employment**			
No	280 (60.7)	259 (58.9)	539 (59.8)
Yes	181 (39.3%)	181 (41.1)	362 (40.2)
**Do you have any debt?**			
No	238 (51.6)	219 (49.8)	457 (50.7)
Yes	223 (48.4)	221 (50.2)	444 (49.3)
**Difficulty meeting day-to-day expenses in the past month**			
No	169 (36.7)	166 (37.7%)	335 (37.2%)
Yes	292 (63.3)	274 (62.3%)	566 (62.8%)
**Slept hungry in the past month due to financial difficulties**			
No	294 (63.8)	261 (59.3)	555 (61.6)
Yes	167 (36.2)	179 (40.7)	346 (38.4)

Data are presented as numbers (percentage)

The majority of the participants presented with first self-harm attempt (*n* = 806, 89.5%). Pesticides were the most common method to attempt self-harm (*n* = 403, 44.7%) (Table 2). A total of 607 (67.4%) participants had clear expectations of a fatal outcome. The majority of the participants 594 (65.9%) did not communicate that they were thinking of self-harm. Similarly, the majority of the participants (*n* = 585, 65.3%) did not communicate self-harm plans to anyone. A total of 556 (61.7%) participants reported a serious/extreme intent to die. Majority of the participants (*n* = 703, 78.0%) reported interpersonal problems as the precipitant of their self-harm episode and 169 (18.8%) stated that they harmed themselves because of financial problems (Table 2).

**Table 2:** Self-harm characteristics of patients by treatment group

	**E-TAU (*N* = 461)**	**CMAP plus E-TAU (*N* = 440)**	**Total (*N* = 901)**
**Suicide attempts**			
1 attempt	411 (89.2)	395 (89.8)	806 (89.5)
2 + attempts	50 (10.8)	45 (10.2)	95 (10.5)
**Self-harm method**			
Pesticide	204 (44.3)	199 (45.2)	403 (44.7)
Ingestion of toxic chemicals	147 (31.9)	144 (32.7)	291 (32.3)
Pesticides plus ingestion of medication	2 (0.4)	4 (0.9)	6 (0.7)
Ingestion of medication	83 (18.0)	72 (16.4)	155 (17.2)
Gunshot	1 (0.2)	0 (0.0)	1 (0.1)
Jumping from heights	3 (0.7)	3 (0.7)	6 (0.7)
Cuts/others	21 (4.6)	18 (4.1)	39 (4.3)
**Subject’s expectation of fatal outcome**			
No expectation	52 (11.3)	37 (8.4)	89 (9.9)
Uncertain of outcome	110 (23.9)	95 (21.6)	205 (22.8)
Clear expectations of fatal outcome	299 (64.9)	308(70.0)	607 (67.4)
**Communication of self-harm intent**			
No	297 (64.4)	297 (67.5)	594 (65.9)
Indirect communication,	22 (4.8)	13 (3.0)	35 (3.9)
Direct communication	142 (30.8)	130 (29.5)	272 (30.2)
**Threaten suicide or did anything that could be or was interpreted by someone else as a threat to harm or kill self**			
No	292 (63.9)	293 (66.7)	585 (65.3)
Indirect threat	26 (5.7)	13 (3.0)	39 (4.4)
Direct threat	139 (30.4)	133 (30.3)	272 (30.4)
**Intent to die**			
Obviously no intent/minimal	69 (15.0)	56 (12.7)	125 (13.9)
Definite intent but very ambivalent	109 (23.6)	111 (25.2)	220 (24.4)
Serious intent/extreme intent	283 (61.4)	273 (62.0)	556 (61.7)
**Precipitants**			
Interpersonal problems	369 (80.0)	334 (75.9)	703 (78.0)
Financial problems	79 (17.1)	90 (20.5)	169 (18.8)
Other	13 (2.8)	16 (3.6)	29 (3.2)

Data are presented as numbers (percentage)

Although there was a trend towards fewer repetitions in the CMAP plus E-TAU arm, there was no statistically significant difference in the proportion of repetition of self-harm between the two arms (intervention — *n* = 17 (3.9%) vs. E-TAU — *n* = 23 (5.1%)) at 12 months. The odds ratio was estimated to be 0.78 (*p*-value = 0.459) (Table 3).

**Table 3:** Repetition rate of self-harm at 12 months by treatment group

	**E-TAU**	**CMAP plus E-TAU**	**Odd ratio **^**a**^** (95% CI)**	***P*-value**
No repetition	429 (94.9)	416 (96.1)		
Repetition	23 (5.1)	17 (3.9)	0.78 (0.41, 1.50)	0.459

Adjusted for age, gender, method of self-harm and depression at baseline

^a^ Expressed as odds for CMAP + E-TAU group relative to odds for E-TAU group

There were a total of 19 adverse events (not related to intervention). A total of nine in the intervention arm (2 = worsening of physical consequences of self-harm, 1 = episode of major depressive disorder, 2 = road accidents, 1 = typhoid, 1 = tuberculosis, 1 = heart disease, and 1 = appendicitis) and ten were in E-TAU (1 = worsening of physical consequences of self-harm, 3 = episode of major depressive disorder, 1 = alcohol dependence, 1 = malaria, 2 = psychosis, 1 = jaundice, and 1 = road accident).

Participants in the intervention arm compared to the E-TAU showed significantly greater improvements on all the key clinical measures correlated with suicide (suicidal ideation, depression, and hopelessness) at 3, 6, 9, and 12 months (except for suicidal ideation and hopelessness at 12 months) (*p* < 0·05). In terms of coping resources, there were statistically significant differences between the two trial arms on overall CRI score as well as on all 4 domains (cognitive, social, spiritual/philosophical, and physical) at each follow-up (except for physical domain at 9-month follow-up) and health-related quality of life at each follow-up (*p* < 0.05) (Table 4).

**Table 4:** Scores for symptom measures at baseline, 3, 6, 9 and 12 months, by treatment group

	**E-TAU**		**CMAP plus E-TAU**		**Mean difference **^**a**^** (95% CI)**	***P*-value**
	***N***	**Mean (SD)**	***N***	**Mean (SD)**		
**Beck Suicide Ideation Scale**						
Baseline	461	10.1 (8.7)	440	10.5 (8.9)		
3 months	446	5.6 (7.7)	425	1.9 (4.9)	− 3.6 (− 4.9, − 2.4)	< .001
6 months	438	3.9 (6.6)	426	1.5 (4.6)	− 2.3 (− 3.6, − 1.1)	< .001
9 months	434	3.2 (5.9)	420	1.4 (4.3)	− 1.7 (− 3.0, − 0.5)	.007
12 months	431	1.9 (5.1)	424	0.9 (3.5)	− 1.0 (− 2.2, 0.3)	.133
**Beck Depression Inventory**						
Baseline	461	25.1 (12.5)	440	25.6 (11.9)		
3 months	446	18.7 (12.7)	425	11.6 (10.7)	− 7.1 (− 8.7, − 5.4)	< .001
6 months	438	14.9 (10.5)	426	9.3 (9.3)	− 5.5 (− 7.2, − 3.9)	< .001
9 months	434	11.9 (9.3)	420	7.8 (8.4)	− 4.1 (− 5.7, − 2.5)	< .001
12 months	431	8.9 (10.5)	424	6.5 (8.7)	− 2.4 (− 4.0, − 0.8)	.004
**Beck Hopelessness Scale**						
Baseline	461	9.5 (6.0)	440	9.5 (6.0)		
3 months	446	7.5 (5.6)	425	4.9 (4.9)	− 2.6 (− 3.4, − 1.8)	< .001
6 months	438	7.1 (5.7)	426	4.2 (4.8)	− 2.9 (− 3.7, − 2.0)	< .001
9 months	434	5.4 (4.9)	420	4.5 (4.6)	− 0.9 (− 1.7, − 0.1)	.034
12 months	431	4.2 (5.2)	424	3.4 (4.4)	− 0.8 (− 1.6, 0.0)	.054
**Coping Resource Inventory (CRI)**						
Baseline	461	150 (28)	440	148 (29)		
3 months	446	154 (2)	425	166 (26)	12 (8, 16)	< .001
6 months	438	158 (26)	426	169 (24)	11 (7, 15)	< .001
9 months	434	163 (25)	420	168 (24)	6 (2, 10)	.002
12 months	431	176 (30)	424	183 (29)	8 (4, 12)	< .001
**CRI: Cognitive Raw Score**						
Baseline	461	22.9 (6.4)	440	22.5 (6.7)		
3 months	446	23.9 (6.4)	425	26.6 (5.6)	2.8 (2.0, 3.6)	< .001
6 months	438	24.5 (6.0)	425	27.0 (5.3)	2.6 (1.8, 3.4)	< .001
9 months	434	26.2 (5.5)	420	27.5 (5.3)	1.3 (0.5, 2.1)	.001
12 months	431	28.3 (6.4)	423	29.7 (5.9)	1.5 (0.7, 2.3)	< .001
**CRI: Social Raw Score**						
Baseline	461	33.2 (7.5)	440	32.6 (7.9)		
3 months	446	34.1 (7.2)	425	37.0 (6.9)	3.0 (2.0, 4.0)	< .001
6 months	438	35.4 (6.5)	426	37.8 (6.6)	2.5 (1.5, 3.5)	< .001
9 months	434	36.6 (6.4)	420	38.2 (6.7)	1.6 (0.6, 2.7)	.002
12 months	431	39.4 (7.7)	424	41.0 (7.6)	1.7 (0.7, 2.7)	.001
**CRI: Emotional Raw Score**						
Baseline	461	39.9 (8.9)	440	39.3 (9.0)		
3 months	446	40.9 (8.6)	425	43.8 (8.1)	3.0 (1.6, 4.3)	< .001
6 months	437	42.2 (8.1)	426	45.2 (7.6)	3.1 (1.7, 4.4)	< .001
9 months	434	43.2 (7.9)	420	44.7 (7.6)	1.6 (0.2, 2.9)	.020
12 months	431	46.9 (9.6)	423	49.2 (9.3)	2.4 (1.1, 3.8)	.001
**CRI: Spiritual/philosophical Raw Score**						
Baseline	461	28.6 (6.1)	440	28.5 (6.1)		
3 months	445	29.3 (6.0)	425	30.9 (5.7)	1.9 (1.0, 2.7)	< .001
6 months	438	30.1 (5.7)	426	31.6 (4.8)	1.8 (0.9, 2.7)	< .001
9 months	434	30.2 (5.3)	420	31.2 (5.2)	1.1 (0.3, 2.0)	.011
12 months	431	33.1 (6.0)	424	34.2 (5.9)	1.4 (0.5, 2.2)	.002
**CRI: Physical Raw Score**						
Baseline	461	25.6 (4.5)	440	25.2 (4.4)		
3 months	446	25.9 (4.9)	425	27.4 (4.6)	1.7 (1.1, 2.3)	< .001
6 months	438	26.3 (4.5)	426	27.7 (4.3)	1.5 (0.9, 2.1)	< .001
9 months	434	26.2 (4.1)	420	26.6 (3.9)	0.5 (− 0.1, 1.0)	.121
12 months	431	28.3 (4.7)	424	29.1 (4.6)	0.9 (0.3, 1.5)	.003
**EQ-5D Index Score**						
Baseline	461	0.48 (0.38)	440	0.46 (0.40)		
3 months	446	0.65 (0.34)	425	0.78 (0.30)	0.13 (0.08, 0.17)	< .001
6 months	438	0.74 (0.28)	426	0.82 (0.26)	0.08 (0.04, 0.12)	< .001
9 months	434	0.79 (0.27)	420	0.86 (0.24)	0.06 (0.02, 0.11)	.005
12 months	431	0.82 (0.29)	424	0.86 (0.22)	0.05 (0.00, 0.09)	.041
**EQ-5D visual analogue scale**						
Baseline	461	54.8 (19.9)	440	53.7 (21.4)		
3 months	446	60.4 (20.2)	425	69.9 (19.6)	9.5 (6.9, 12.1)	< .001
6 months	438	64.8 (19.4)	426	75.0 (17.1)	10.2 (7.6, 12.8)	< .001
9 months	434	67.9 (17.5)	420	74.2 (15.6)	6.3 (3.7, 8.9)	< .001
12 months	431	73.0 (18.5)	424	75.6 (17.8)	2.6 (− 0.0, 5.2)	.054

Adjusted for age, gender, method of self-harm and depression at baseline

^a^ Expressed as outcome for CMAP + E-TAU group minus outcome for E-TAU group

All CSQ-8 outcomes were significantly higher in the intervention arm compared to the E-TAU (all *p* < 0.001). The quality of services was rated as good to excellent by 385 (90.6%) participants in the intervention arm compared to 344 (77.1%) participants in the E-TAU arm (Table 5).

**Table 5:** Comparison between E-TAU arm and CMAP plus E-TAU arm on the CSQ-8 at 3-month FU

Client Satisfaction Questionnaire CSQ-8	E-TAU	CMAP plus E-TAU	Odds ratio ^a^ (95% CI)	*P*-value
**Q1. How would you rate the quality of service you received?**				
Poor	8 (1.8)	2 (0.5)	2.83	< .001
Fair	94 (21.1)	38 (8.9)	(2.01, 3.98)	
Good	244 (54.7)	203 (47.8)		
Excellent	100 (22.4)	182 (42.8)		
**Q2. Did you get the kind of service you wanted?**				
No, definitely not	25 (5.6)	2 (0.5)	3.66	< .001
No, not really	31 (7.0)	6 (1.4)	(2.364, 5.66)	
Yes, generally	224 (50.2)	148 (34.8)		
Yes, definitely	166 (37.2)	269 (63.3)		
**Q3. To what extent has our service met your needs?**				
None of my needs have been met	39 (8.7)	5 (1.2)	3.85	< .001
Only a few of my needs have been met	150 (33.6)	73 (17.2)	(2.63, 5.64)	
Most of my needs have been met	200 (44.8)	206 (48.5)		
Almost all of my needs have been met	57 (12.8)	141 (33.2)		
**Q4. If a friend were in need of similar help, would you recommend our service to him or her?**				
No, definitely not	6 (1.3)	3 (0.7)	1.99	< .001
No, I don’t think so	10 (2.2)	3 (0.7)	(1.40, 2.84)	
Yes, I think so	184 (41.3)	124 (29.2)		
Yes, definitely	246 (55.2)	295 (69.4)		
**Q5. How satisfied are you with the amount of help you received?**				
Quite dissatisfied	6 (1.3)	2 (0.5)	3.15	< .001
Indifferent or mildly dissatisfied	58 (13.0)	21 (4.9)	(2.194, 4.51)	
Mostly satisfied	263 (59.0)	189 (44.5)		
Very satisfied	119 (26.7)	213 (50.1)		
**Q6. Have the services you received helped you to deal more effectively with your problems?**				
No, they seemed to make things worse	6 (1.3)	0 (0.0)	3.89	< .001
No, they really didn’t help	44 (9.9)	15 (3.5)	(2.65, 5.72)	
Yes, they helped somewhat	299 (67.0)	199 (46.8)		
Yes, they helped a great deal	97 (21.7)	211 (49.6)		
**Q7. In an overall, general sense, how satisfied are you with the service you received?**				
Quite dissatisfied	10 (2.2)	4 (0.9)	3.60	< .001
Indifferent or mildly dissatisfied	58 (13.0)	18 (4.2)	(2.494, 5.21)	
Mostly satisfied	285 (63.9)	207 (48.7)		
Very satisfied	93 (20.9)	196 (46.1)		
**Q8. If you were to seek help again, would you come back to our service?**				
No, definitely not	5 (1.1)	3 (0.7)	2.43	< .001
No, I don’t think so	17 (3.8)	6 (1.4)	(1.65, 3.57)	
Yes, I think so	181 (40.6)	106 (24.9)		
Yes, definitely	243 (54.5)	310 (72.9)		

Data are presented as number (percentage)

Adjusted for age, gender, method of self-harm and depression at baseline

^a^ Expressed as odds for CMAP + E-TAU group relative to odds for E-TAU group

Moreover, the session attendance log showed that 413 (93·87%) participants in the intervention arm attended 5 to 6 sessions.

The fidelity ratings of all therapists were satisfactory and ranged between 4 and 6 on 12 items of CTRs. A rating of 4 indicates “good features, but minor problems and/or inconsistencies”. A rating of 6 indicates “excellent performance, even in the face of patient difficulties” (Table 6).

**Table 6:** Fidelity rating on CTRS (*n* = 25 therapists)

Items/domains on CTRS	Minimum	Maximum	Mean	Std. deviation
Agenda setting and adherence	4	6	4.92	.493
Feedback	4	6	4.76	.523
Collaboration	4	6	4.88	.440
Pacing and efficient use of time	4	6	4.96	.351
Interpersonal effectiveness	4	6	5.04	.351
Eliciting of appropriate emotional expression	4	5	4.72	.458
Eliciting key cognitions	4	5	4.92	.277
Eliciting and planning behaviours	4	5	4.76	.436
Guided discovery	4	5	4.72	.458
Conceptual integration	4	5	4.52	.510
Application of change methods	4	5	4.72	.458
Homework setting	5	6	5.52	.510

## Discussion

The CMAP trial is one of the few trials which evaluated a CBT-based culturally adapted psychological intervention to reduce self-harm and clinical outcomes known to be predictive of suicide and the first trial of its kind in any low- and middle-income country (LMIC). This trial showed that the repetition rate of self-harm was low for both groups at 12 months, although the number in the intervention arm was lower (*n* = 17) compared to the E-TAU arm (*n* = 23), but the difference between the two arms was not statistically significant. There was a significant reduction in the intervention arm compared to the E-TAU arm in suicidal ideation, depression, and hopelessness at the end of intervention. Similarly, intervention arm participants reported significantly better health-related QoL and better coping skills compared to the E-TAU arm.

Consistent with our findings, a previous trial investigating the effectiveness of Volitional Help Sheets also did not report any statistically significant differences both in terms of repetition (67 intervention vs 71 TAU) and suicide rate (one in intervention vs two in TAU) [34]. For the current trial, there is a disparity between the repetition rate in Brown et al., the study used for sample size calculation, and that observed in this trial. The possible reason for this disparity in expected and observed event rate could be that Brown et al., study was conducted in a high-income country (Philadelphia) [33] and despite a different setting our sample size calculation was based on this study because of lack of evidence on the self-harm repetition rate in Pakistan and also the lack of evidence on therapist-delivered intervention trials to prevent the repetition of self-harm both in Pakistan and in other similar low-income settings. Assuming the exact event rates as per the observed data in current trial (5.1% and 3.9%) in the two groups, this is only a small difference of 1.2%. For a 5% significance level and 80% power a sample of 4684 per group, 9368 would be required in total. For a clinically meaningful difference of 2% a sample of 3372 in total would be required. Moreover, the low repetition rate in the E-TAU group in this study is supported by a recent meta-analysis of 14 studies on brief interventions delivered in a single encounter (such as brief follow-up contacts and safety planning) to those at high risk of suicide are effective at improving outcomes (such as subsequent suicide attempts) [35]. The participants in the E-TAU arm in current trial received comprehensive health assessments along with a monthly call by researchers to maintain engagement which may have had a therapeutic effect [35].

The majority of the participants in the trial reported that they had serious intent to die. In a recent report, of those who presented to hospitals with suicidal ideation, the risk of self-harm within 12 months was 10% and 18% within 5 years [36]. In the current trial participants in both study arms were in a high-risk group (score greater than six on Beck Suicide Ideation scale) at baseline and for both arms there was a reduction in suicidal ideation at each follow-up point. However, this was significantly greater in the intervention arm compared to the E-TAU arm and participants in the intervention arm were no longer in a high-risk category at the 3-month follow-up and this trend was sustained till 12-month follow-up, and those in the E-TAU arm did not achieve non-risk category until 12-month follow-up. Though the mean difference between two groups was not statistically significant at 12-month follow-up, both groups were in the non-risk category. An exploratory trial of CMAP also showed a sustained effect of CMAP on suicidal ideation at 6-month follow-up [5]. Participants in E-TAU arm achieving non-risk category on suicidal ideation is also supported by a published trial that showed a significant reduction in scores on the suicidal ideation scale at 6-month follow-up after participation in a low-intensity intervention called motivational interviewing [37].

Suicide theories such as Interpersonal–Psychological Theory of Suicide [38] and Hopelessness Theory of Suicide [39] incorporate hopelessness and depression as potential causes of suicidal thoughts or behaviours. Therefore, the management of depression and hopelessness are likely important mitigating factors in self-harm and suicide prevention. In the current trial, depression was reduced for both groups at each follow-up, however, mean scores were significantly lower in the intervention arm compared to the E-TAU arm. Moreover, participants in the intervention arm achieved remission (score < 13) earlier (at 3-month follow-up) compared to the E-TAU arm (at 9-month follow-up). Hopelessness scores were reduced for both at all follow-ups, however, mean scores were significantly lower in the intervention arm compared to the E-TAU arm at 3-, 6- and 9-month follow-ups. Though the mean difference between the two groups was not statistically significant at 12-month follow-up, both groups were in the non-risk (minimal to mild) hopelessness category. Evidence shows that CBT-based interventions have a beneficial effect on depression and hopelessness [10] with few trials showing a sustained effect of interventions at 12-month follow-up (9-h-long sessions of problem-solving intervention over 3 months) [40], (10-session CBT intervention) [33], and (5 sessions of problem-solving intervention within 1 month of index self-harm attempt) [41]. This may indicate that a long-term impact on hopelessness may require either more intervention sessions or more frequent sessions immediately after a self-harm attempt. Future trials may also consider evaluating the role of booster sessions [34].

The nature of the stressors that trigger self-harm behaviours may be difficult to change, but the coping strategies to deal with stressors are dynamic and amenable to change. Therefore, strengthening coping resources can be a helpful strategy to reduce self-harm behaviours [42]. Findings from the current trial show that participants in the intervention arm reported significantly better coping resources at each follow-up compared to the E-TAU arm for all domains (cognitive, social, emotional, and spiritual) except physical coping where the difference between the two arms was not significant at 9- and 12-month follow-up.

Mental health interventions may not only reduce suicidal behaviours but may also contribute in improving QoL [5]. The current trial shows that participants in both arms improved but those who received the intervention had significantly better QoL compared to E-TAU at each follow-up. Findings are consistent with earlier CMAP trials [5]. This also highlights the importance of assessment of QoL of those who are at risk of suicide through simple easy to administer tools such as EQ-5D [5].

### Strengths and limitations

To the best of our knowledge, this was the largest therapist-delivered self-harm and suicide prevention trial across the world, with a high retention rate at a long-term follow-up. However, in most low-income settings (including Pakistan) access to trained mental health professionals/therapists is limited particularly in rural settings, telehealth solutions can address such challenges related to access. Mental health professionals in these settings may also consider how best to engage and train other allied health professionals such as nurses, and community health workers in the delivery of suicide prevention interventions or components of these interventions. Participants were recruited from a variety of settings (including primary care settings) across Pakistan, increasing the likelihood of the generalizability of findings. Moreover, the use of a detailed semi-structured tool (SASII) to assess the primary outcome, structured validated instruments to assess secondary outcomes, regular training, rigorous arrangements for supervision, and fidelity assessment have increased the validity of trial findings. In addition, a large sample size, with long-term follow-up is also a strength of the trial. However, since the risk of self-harm in those with suicidal ideation increases with time, therefore a longer-term follow-up is recommended. There was a disparity between the self-harm repetition rate expected based on sample size calculation and the repetition rate observed in the trial. A trial with an even larger sample size would be required to detect such a small difference observed in this study. Moreover, the CMAP intervention focused on different components that are likely to reduce social stigma and improve the awareness of self-harm and its prevention such as psychoeducation about the motivations behind self-harm episodes, emotional consequences of the episode, importance of seeking professional help etc. Future trials of CMAP intervention may consider assessing change in participants’ attitude and behaviours towards self-harm and suicide at end of intervention.

Further research is also needed to explore the role of brief follow-up contact, such as using postcards in low resource settings. The clinical staging model which involves multiple intervention stages have been found to be effective [43] and may also be helpful in suicide prevention. These are interventions in which the type or dosage is individualised based on patient characteristics (such as clinical presentations) and is repeatedly adjusted in response to the individual’s progress.

## Conclusions

CMAP intervention is promising for improving clinical outcomes predictive of suicide, coping resources, health-related QoL, and perceived service quality among adult self-harm survivors in Pakistan. All individuals who participated in the trial reported low repetition rates and there were low suicide rates. These findings on the role of brief interventions and enhanced usual care in improving outcomes predictive of self-harm and suicide (hopelessness, suicidal ideation and depression) are particularly important for low-resource settings where delivering more resource-intensive interventions is challenging.

## Data Availability

Anonymised data will be made available on request.

## Abbreviation

LMICs: Low- and middle-income countries
CMAP: Culturally adapted manual-assisted problem-solving intervention
E-TAU: Enhanced treatment as usual
CBT: Cognitive behaviour therapy
QoL: Quality of life
WHO: World Health Organisation
RCTs: Randomised controlled trials
DBT: Dialectical behaviour therapy
MBT: Mentalisation-based therapy
NICE: National Institute for Health and Care Excellence
CONSORT: Consolidated Standards of Reporting Trials
ID: Identification number
SASII: Suicide Attempt Self-Injury Interview
BSI: Beck Scale for Suicide ideation
BHS: Beck Hopelessness Scale
BDI: Beck Depression Inventory
CRI: Coping resource inventory
EQ-5D: EuroQol – 5 Dimensions
CSQ: Client Satisfaction Questionnaire
CTRS: Cognitive Therapy Rating Scale
GCP: Good Clinical Practice
ICC: Intra-cluster correlation coefficient

## References

[1] WHO WHO. Suicide worldwide in 2019: global health estimates. 2021. https://www.who.int/publications/i/item/9789240026643.

[2] WHO WHO. Suicide Data. 2023. https://www.who.int/news-room/fact-sheets/detail/suicide.

[3] Vijayakumar L, Daly C, Arafat Y, Arensman E. Suicide prevention in the Southeast Asia region. Crisis. 2020. 10.1027/0227-5910/a000666. 10.1027/0227-5910/a000666 32208757

[4] Patel V, Ramasundarahettige C, Vijayakumar L, Thakur J, Gajalakshmi V, Gururaj G (2012). Suicide mortality in India: a nationally representative survey. Lancet.

[5] Husain N, Afsar S, Ara J, Fayyaz H, Ur Rahman R, Tomenson B (2014). Brief psychological intervention after self-harm: randomised controlled trial from Pakistan. BJPsych.

[6] TRIBUNE TE. Law penalising suicide attempts abolished. 2023. https://tribune.com.pk/story/2392477/law-penalising-suicide-attempts-abolished.

[7] Kiran T, Chaudhry N, Bee P, Tofique S, Farooque S, Qureshi A (2021). Clinicians’ perspectives on self-harm in Pakistan: a qualitative study. Front Psychiatry.

[8] Pirani S, Qureshi A, Khan MZ, Aslam M, Khan MM (2023). Assessing knowledge, attitudes, and practices of emergency department staff towards patients with suicidal behaviors in Pakistan. Asian J Psychiatry.

[9] Nawaz RF, Reen G, Bloodworth N, Maughan D, Vincent C (2021). Interventions to reduce self-harm on in-patient wards: systematic review. BJPsych Open.

[10] Witt KG, Hetrick SE, Rajaram G, Hazell P, Salisbury TLT, Townsend E (2021). Psychosocial interventions for self-harm in adults. Cochrane Database Syst Rev.

[11] Westbrook D, Kennerley HJK (2008). An introduction to cognitive behaviour therapy: skills and applications. An introduction to cognitive behaviour therapy: skills and applications.

[12] NICE NIfHCE (2022). Self-harm: assessment, management and preventing recurrence.

[13] Schmidt U, Davidson K (2004). Life after self-harm: A guide to the future.

[14] Fleischmann A, Bertolote JM, Wasserman D, De Leo D, Bolhari J, Botega NJ (2008). Effectiveness of brief intervention and contact for suicide attempters: a randomised controlled trial in five countries. Bull World Health Organ.

[15] Schmidtke A, Bille-Brahe U, DeLeo D, Kerkhof A, Bjerke T, Crepef P (1996). Attempted suicide in Europe: rates, trend. S and sociodemographic characteristics of suicide attempters during the period 1989–1992. Results of the WHO/EURO multicentre study on parasuicide. Acta Psychiatr Scand.

[16] Juul S, Gluud C, Simonsen S, Frandsen FW, Kirsch I, Jakobsen JC (2021). Blinding in randomised clinical trials of psychological interventions: a retrospective study of published trial reports. BMJ Evid - Based Med.

[17] Linehan MM, Comtois KA, Brown MZ, Heard HL, Wagner A (2006). Suicide Attempt Self-Injury Interview (SASII): development, reliability, and validity of a scale to assess suicide attempts and intentional self-injury. Psychol Assess.

[18] Beck AT, Steer RA (1991). BSI, Beck scale for suicide ideation: manual.

[19] De Beurs DP, Fokkema M, O’Connor RC (2016). Optimizing the assessment of suicidal behavior: the application of curtailment techniques. J Affect Disord.

[20] Husain MO, Umer M, Taylor P, Chaudhry N, Kiran T, Ansari S (2019). Demographic and psychosocial characteristics of self-harm: the Pakistan perspective. Psychiatry Res.

[21] Beck A, Steer R (1988). Manual for the Beck hopelessness scale.

[22] Beck AT, Ward CH, Mendelson M, Mock J, Erbaugh J (1961). An inventory for measuring depression. Arch Gen Psychiatry.

[23] Hammer AL, Marting MS (1988). Manual for the coping resources inventory.

[24] Brooks R, Group E (1996). EuroQol: the current state of play. Health Policy.

[25] Kangwanrattanakul K, Parmontree P (2020). Psychometric properties comparison between EQ-5D-5L and EQ-5D-3L in the general Thai population. Qual Life Res.

[26] Abdin E, Chong SA, Seow E, Peh CX, Tan JH, Liu J (2019). A comparison of the reliability and validity of SF-6D, EQ-5D and HUI3 utility measures in patients with schizophrenia and patients with depression in Singapore. Psychiatry Res.

[27] Attkisson CC, Greenfield TK. The UCSF Client Satisfaction Scales: I. The Client Satisfaction Questionnaire-8. In M. E. Maruish (Ed.), The use of psychological testing for treatment planning and outcomes assessment: Instruments for adults. Lawrence Erlbaum Associates Publishers; 2004. pp. 799–811.

[28] Attkisson CC, Greenfield TK (1996). The client satisfaction questionnaire (CSQ) scales and the service satisfaction scale-30 (SSS-30). Outcomes Assess Clin Pract.

[29] Blackburn I-M, James IA, Milne DL, Baker C, Standart S, Garland A (2001). The revised cognitive therapy scale (CTS-R): psychometric properties. Behav Cognitive Psychother.

[30] Goldberg SB, Baldwin SA, Merced K, Caperton DD, Imel ZE, Atkins DC (2020). The structure of competence: evaluating the factor structure of the cognitive therapy rating scale. Behav Ther.

[31] Beecham J, Knapp M (2001). Costing psychiatric interventions. Meas Mental Health Needs.

[32] Roberts C, Roberts SA (2005). Design and analysis of clinical trials with clustering effects due to treatment. J Clin Trials.

[33] Brown GK, Ten Have T, Henriques GR, Xie SX, Hollander JE, Beck AT (2005). Cognitive therapy for the prevention of suicide attempts: a randomised controlled trial. JAMA.

[34] O'Connor RC, Ferguson E, Scott F, Smyth R, McDaid D, Park A-L (2017). A brief psychological intervention to reduce repetition of self-harm in patients admitted to hospital following a suicide attempt: a randomised controlled trial. Lancet Psychiat.

[35] Doupnik SK, Rudd B, Schmutte T, Worsley D, Bowden CF, McCarthy E (2020). Association of suicide prevention interventions with subsequent suicide attempts, linkage to follow-up care, and depression symptoms for acute care settings: a systematic review and meta-analysis. JAMA Psychiat.

[36] Griffin E, Kavalidou K, Bonner B, O'Hagan D, Corcoran P (2020). Risk of repetition and subsequent self-harm following presentation to hospital with suicidal ideation: A longitudinal registry study. EClinicalMedicine.

[37] Britton PC, Conner KR, Chapman BP, Maisto SA (2020). Motivational interviewing to address suicidal ideation: a randomised controlled trial in veterans. Suicide Life-Threat Behav.

[38] Van Orden KA, Witte TK, Cukrowicz KC, Braithwaite SR, Selby EA, Joiner TE (2010). The interpersonal theory of suicide. Psychol Rev.

[39] Abramson LY, Alloy LB, Hogan ME, Whitehouse WG, Gibb BE, Hankin BL (2002). The hopelessness theory of suicidality.

[40] Hatcher S, Sharon C, Parag V, Collins N (2011). Problem-solving therapy for people who present to hospital with self-harm: Zelen randomised controlled trial. BJPsyc.

[41] Salkovskis PM, Atha C, Storer D (1990). Cognitive-behavioural problem solving in the treatment of patients who repeatedly attempt suicide a controlled trial. BJPsyc.

[42] Gelinas BL, Wright KD (2013). The cessation of deliberate self-harm in a university sample: The reasons, barriers, and strategies involved. Arch Suicide Res.

[43] McGorry PD, Hartmann JA, Spooner R, Nelson B (2018). Beyond the “at risk mental state” concept: transitioning to transdiagnostic psychiatry. World Psychiatry.

